# Physician global assessment in rheumatoid arthritis—is there any logic?

**DOI:** 10.1093/rap/rkaf139

**Published:** 2025-12-03

**Authors:** Katriina Jäppinen, Paula Muilu, Reija Autio, Laura Kuusalo, Susanna Sihvonen, Tuulikki Sokka-Isler, Vappu Rantalaiho

**Affiliations:** Faculty of Medicine and Health Technology, Tampere University, Tampere, Finland; Centre for Rheumatic Diseases, Tampere University Hospital, Tampere, Finland; Health Sciences, Faculty of Social Sciences, Tampere University, Tampere, Finland; Rheumatology, University of Turku and Turku University Hospital, Turku, Finland; Centre for Rheumatic Diseases, Tampere University Hospital, Tampere, Finland; Faculty of Heath Sciences, University of Eastern Finland, Kuopio, Finland; Hospital Nova, Wellbeing Services County of Central Finland, Jyväskylä, Finland; Faculty of Medicine and Health Technology, Tampere University, Tampere, Finland; Centre for Rheumatic Diseases, Tampere University Hospital, Tampere, Finland; Kanta-Häme Central Hospital, Hämeenlinna, Finland

Key message

- A standardised, internationally validated approach to the Physician Global Assessment in RA could be considered to ensure reliable registry data.

Dear Editor, Untreated, rheumatic diseases lead to tissue destruction, premature disability and reduced life expectancy. Currently, disease activity is assessed based on joint counts, inflammatory markers, patient-reported outcome measures (PROMs) and imaging [1]. The Physician Global Assessment (PGA) is used to evaluate overall disease activity in many rheumatic diseases. At its best, it reflects the physician’s interpretation of symptoms attributable to disease activity, excluding symptoms caused by comorbidities or previous lesions. Furthermore, PGA has been shown to predict treatment failure and to correlate with objective measures of disease activity in rheumatoid arthritis (RA) [2, 3]. However, while the general method has been described, no modern standardized guidelines exist for the assessment of PGA [4]. Also, the studies on variability of PGA in RA remain limited [5].

This is clinically important, since PGA relies on individual physician judgement and experience. We investigated how Finnish rheumatologists assess PGA in RA and whether the scoring variability warrants a formal validation process, as has been initiated by paediatric rheumatologists [6, 7].

At the Finnish Society for Rheumatology’s convention in January 2024, 75 physicians reviewed 16 anonymised RA patient cases representing a broad spectrum of disease activity. Each case included a short clinical history, disease activity parameters, PROMs and joint counts (Supplementary Table S1, available at *Rheumatology Advances in Practice* online). The physicians rated PhGA on a 0–10 scale, and evaluated the importance of different factors affecting their PhGA on a 0–5 scale (Table 1). All participating physicians provided informed consent to attend the trial. All data analyses were performed without personal identification data; therefore, ethical approval was not required.

**Table 1.** Importance of different factors physicians take into consideration when assessing PhGA

	All physicians						
Factors physicians take into consideration and their importance (on a scale from 0 to 5)	Mean (SD)	Range	Group A (*n* = 8) mean (SD)	Group B (*n* = 7) mean (SD)	Group C (*n* = 60) mean (SD)	*P*-value^a^	Adjusted *P*-value
Swollen joint count (SJC)	4.75 (0.50)	3.00–5.00	4.75 (0.46)	5.00 (0.00)	4.72 (0.52)	0.330	0.424
Size of swollen joints	2.89 (1.15)	0.00–5.00	3.00 (0.93)	2.86 (1.07)	2.88 (1.19)	0.967	0.967
Inflammatory markers	3.60 (0.97)	1.00–5.00	3.88 (0.83)	3.14 (1.21)	3.62 (0.96)	0.416	0.499
DAS28	2.55 (1.24)	0.00–5.00	3.75 (1.04)	0.86 (0.90)	2.59 (1.08)	<0.001	<0.001
Ultrasound findings	4.04 (0.94)	2.00–5.00	4.63 (0.52)	4.57 (0.79)	3.89 (0.96)	0.033	0.054
Tender joint count (TJC)	2.15 (1.18)	0.00–5.00	2.63 (1.30)	1.14 (0.90)	2.20 (1.15)	0.030	0.054
Patient’s pain VAS	1.39 (0.97)	0.00–4.00	2.00 (0.53)	0.29 (0.49)	1.43 (0.96)	0.001	0.004
Patient Global Assessment (PGA)	1.41 (0.89)	0.00–3.00	2.13 (0.64)	0.29 (0.49)	1.45 (0.83)	<0.001	<0.001
Essential change from the previous PGA	1.60 (1.15)	0.00–4.00	2.50 (1.20)	1.00 (1.29)	1.55 (1.08)	0.030	0.054
Active findings in imaging	3.20 (1.27)	0.00–5.00	3.88 (0.99)	2.43 (1.51)	3.20 (1.24)	0.122	0.169
HAQ	1.68 (1.09)	0.00–4.00	2.88 (0.83)	0.71 (0.49)	1.63 (1.04)	0.001	0.005
Patient’s work ability	1.46 (1.11)	0.00–4.00	2.25 (0.71)	0.57 (0.79)	1.46 (1.12)	0.010	0.036
Essential change from the previous HAQ	1.96 (1.30)	0.00–5.00	2.88 (1.25)	1.29 (1.98)	1.92 (1.17)	0.028	0.054
Medication	1.80 (1.19)	0.00–4.00	2.50 (0.76)	1.14 (1.07)	1.78 (1.22)	0.077	0.116
Convincing history of an active disease	2.13 (1.10)	0.00–4.00	3.00 (1.07)	1.57 (0.79)	2.08 (1.08)	0.028	0.054
Non-inflammatory explanation for the symptoms	3.73 (1.22)	0.00–5.00	3.50 (1.60)	4.14 (0.90)	3.72 (1.21)	0.693	0.734
Morning stiffness	2.17 (0.94)	0.00–4.00	2.88 (0.64)	1.57 (0.79)	2.15 (0.94)	0.016	0.048
Overall impression	3.05 (1.15)	0.00–5.00	3.50 (1.07)	2.86 (1.57)	3.02 (1.11)	0.448	0.504

The factors physicians rated on a scale from 0 (not important at all) to 5 (very important) in the online voting in terms of their importance when assessing the Physician Global Assessment (PhGA). The physicians were divided into three groups according to the PhGA values they assigned to the sample patients. Physicians in Group A gave exceptionally high PhGA values to at least two patients with a non-inflammatory explanation for their symptoms. Group B included physicians who never assigned a PhGA value higher than 5. Group included the remaining physicians who were thus considered to recognise the rheumatic condition from a non-rheumatic one and to use the entire VAS when assessing the PhGA.

a The values between the three groups were compared with Kruskal–Wallis test.

*P*-values were multiple adjusted with the Benjamini–Hochberg method. *P*-values <0.05 were considered statistically significant.

SJC: number of swollen joints; DAS28: Disease Activity Score assessing 28 joints; TJC: number of tender joints; VAS: visual analogue scale; PGA: Patient Global Assessment; HAQ: Health Assessment Questionnaire measuring patient’s functional capacity.

Of the participants, 77% were female, and the largest subgroup (39%) had over 10 years of rheumatology experience (Supplementary Table S2, available at *Rheumatology Advances in Practice* online). Overall agreement of the given PhGA-values among the physicians was good (ICC = 0.784, *P* < 0.001); however, the variability was wide in cases without comorbid conditions compared with cases with osteoarthrosis or chronic pain. One case with palindromic RA and no current disease activity received a PhGA score of 0 from all the physicians, improving overall agreement (Supplementary Figs S1 and S2, available at *Rheumatology Advances in Practice* online).

The most important factors influencing PhGAs were the number of swollen joints (SJC), inflammatory markers, ultrasound (US) findings, and potential non-inflammatory explanations for symptoms, while the least important were the patient’s reported visual analogue scale (VAS) for pain, Patient General Assessment (PGA), patient’s work ability, and an essential change from previous PGA (Table 1). We identified three subgroups of physicians. Group A (*n* = 8) gave high (>Q3) PhGA values to at least 2/4 patient cases with non-inflammatory explanations for their symptoms and emphasised significantly more PROMs. Group B (*n* = 7) never assigned a PhGA higher than 5 and placed little weight on PROMs. Group C (*n* = 60) included the remaining physicians who were considered to focus on inflammatory activity and used the entire VAS scale. Their assessments aligned more closely with Group B than A, reflecting overall agreement. The most significant differences between these groups were in the disease activity score assessing 28 joints (DAS28) and PGA. Since rheumatic diseases today tend to present with milder symptoms than in the past, this could have contributed to lower scores given by experienced physicians. Somewhat surprisingly, however, physicians’ experience or gender did not substantially influence PhGA assessment or factors considered important (data not shown). There were no statistically significant differences between the groups in terms of gender and experience; however, the distributions are presented in Supplementary Table S2, available at *Rheumatology Advances in Practice* online.

These findings suggest that while remission and non-inflammatory conditions are generally well recognized, considerable variability exists in how RA activity is assessed using PhGA. This variability may affect clinical decision-making, especially if the treating physicians change, and may also compromise the reliability of registry data. Since there are no modern internationally agreed-upon criteria guiding PhGA assessments, the observed variability appears to reflect individual rheumatologists’ personal approaches, with each clinician developing their own evaluation framework.

According to the attending rheumatologists, US was a significant factor in assessing PhGA. The increasing use of US has changed the field of rheumatology by helping to distinguish non-inflammatory from inflammatory findings, which indeed could reduce the variability of PhGA scoring [6]. Although the availability and use of US undoubtedly vary significantly across countries, its inclusion in joint assessment criteria may be worth considering.

A limitation of our study is the use of paper-based, hypothetical cases, presented in a congress setting. Consequently, the absence of real-life patient interaction limits the broader applicability of our findings. Nevertheless, we hope this study will stimulate further research and international dialogue on the need of broader validation of PhGA in adult rheumatology, as has been initiated by paediatric rheumatologists [7, 8]. Standardized guidelines could improve consistency in clinical care and enhance the reliability of research data.

## Declaration of generative AI and AI-assisted technologies in the writing process

During the preparation of this work, one author (K.J.) used ChatGPT, developed by OpenAI, in order to check the grammar to improve language and readability. After using this tool, the author reviewed and edited the content as needed and takes full responsibility for the content of the publication.

## Supplementary Material

rkaf139_Supplementary_Data

## Data Availability

The data underlying this article will be shared on reasonable request to the corresponding author.
