# Supplementary material for: Physician global assessment in rheumatoid arthritis—is there any logic?
Source: Rheumatol Adv Pract. 2025 Dec 3;9(4):rkaf139. doi: 10.1093/rap/rkaf139 (PMC12684707; doi:10.1093/rap/rkaf139)
Supplement: rkaf139_Supplementary_Data [file rkaf139_supplementary_data.docx]

| **Patient** | **History** | **TJC** | **SJC** | **ESR (mm/h)** | **CRP (nmol/l)** | **DAS28-CRP(3)** | **pain VAS (mm)** | **HAQ** | **PGA (mm)** |
| --- | --- | --- | --- | --- | --- | --- | --- | --- | --- |
| **1** | 40–50 yo female, sporty, seropositive RA 20 years | 4/46 | 1/46 | 10 | 3 | 2.7 | 29 | 0 | 17 |
| **2** | 75 yo male, seropositive RA 15 years | 2/46 | 2/46 | 16 | 7 | 3.9 | 11 | 0.13 | 57 |
| **3** | 55–60 yo female, breast cancer treated, arthrodesis to other ankle done, seropositive RA 15 years | 4/46 | 2/46 | 6 | 1 | 2.7 | 25 | 0.88 | 9 |
| **4** | 60 yo female, seropositive RA 30 years, chronic pain despite longstanding remission on bDMARDs, now new malignancy and planned change of medication | 0/46 | 0/46 | 10 | 2 | 1.6 | 67 | 0.25 | 70 |
| **5** | Patient 4 six months later | 7/46 | 2/46 | 9 | 5 | 3.4 | 66 | 0.38 | 65 |
| **6** | 65 yo female, seropositive RA 40 years, bDMARD 20 years. Now failure after COVID, new bDMARD | 13/46 | 13/46 | 33 | 23 | 5.6 | 37 | 1.25 | 49 |
| **7** | Patient 6 control visit | 7/46 | 7/46 | 29 | 12 | 4.5 | 34 | 1.25 | 35 |
| **8** | 45 yo female, palindromic seropositive RA 15 years, clear history of arthritis activation, now in remission | 0/46 | 0/46 | 5 | 1 | 1.4 | 37 | 0.63 | 37 |
| **9** | Patient 8 five years later on bDMARDs | 0/46 | 0/46 | 8 | 1 | 1.4 | 0 | 0.00 | 0 |
| **10** | 75 yo female, seropositive RA 20 years in remission on bDMARDs, hip osteoarthritis | 2/46 | 1/46 | 2 | 1 | 2.3 | 62 | 0.5 | 47 |
| **11** | 40 yo female, new diagnosis of seropositive RA | 21/46 | 8/46 | 21 | 11 | 4.8 | 78 | 1.5 | 55 |
| **12** | Patient 11 three years later after second bDMARD failure | 4/46 | 2/46 | 16 | 3 | 3.4 | 56 | 1.13 | 49 |
| **13** | 35 yo female, arthralgia, high seropositivity, chronic pain | 1/46 | 0/46 | 17 | 7 | 2.6 | 5 | 0.0 | 30 |
| **14** | Patient 13 three months later, suspected shoulder arthritis | 2/46 | 0/46 | 24 | 6 | 2.8 | 100 | 1.13 | 50 |
| **15** | 70 yo female, seropositive erosive RA 20 years, several join destructions | 4/46 | 4/46 | 20 | 17 | 4.1 | 66 | 1.38 | 53 |
| **16** | 60 yo female, seropositive RA 10 years, no compliance, no effective medication | 2/46 | 27/46 | 61 | 22 | 4.6 | 88 | 2.13 | 81 |

Supplementary Table S1. Sixteen rheumatoid arthritis (RA) patient cases presented at the convention of the Finnish Society for Rheumatology. The attending physicians gave their Physician Global Assessment (PhGA) scores for these cases through online voting. The cases were based on real patients and represented as wide a variety of RA patients as possible. TJC: number of tender joints; SJC: number of swollen joints; ESR: erythrocyte sedimentation rate; CRP: C-reactive protein; DAS28: Disease Activity Score assessing 28 joints; VAS: visual analogue scale; HAQ: Health Assessment Questionnaire measuring patient’s functional capacity; PGA: Patient Global Assessment; yo: years old; bDMARD: biological disease-modifying anti-rheumatic drug.


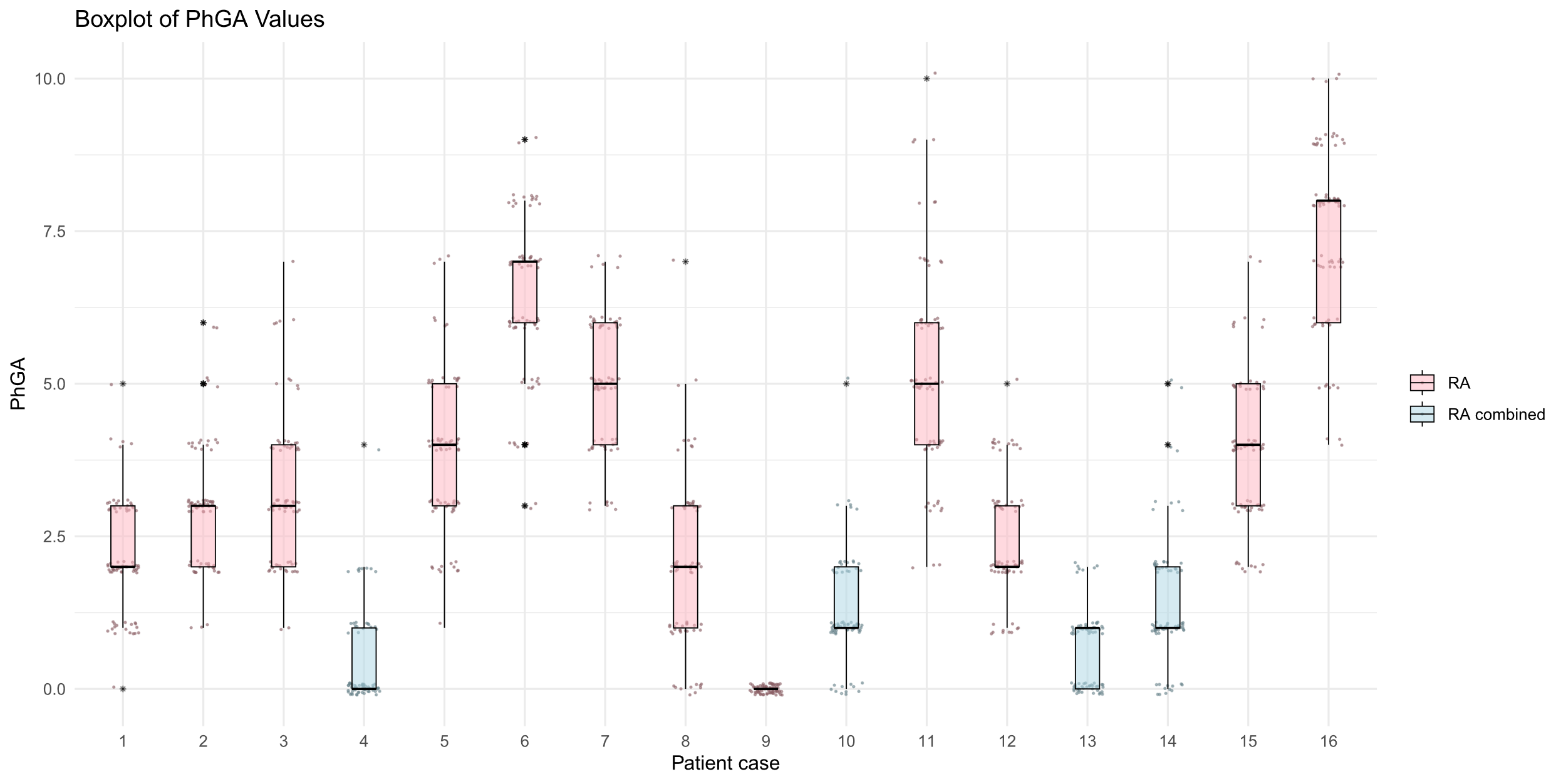


Supplementary Figure S1. Sixteen imaginary rheumatoid arthritis (RA) patient cases were presented to physicians, who assessed the Physician Global Assessment (PhGA) score (on a scale from 0 to 10 in whole numbers, with 0 being no disease activity and 10 being the most severe disease activity) for each case using an online voting system. The boxplot illustrates the distribution of PhGA values across the cases with individual data points showing the variability of the values. The pink colour represents patient cases with RA as their only disease manifestation, while the blue indicates patients with RA combined with non-inflammatory conditions, such as osteoarthritis or chronic pain.


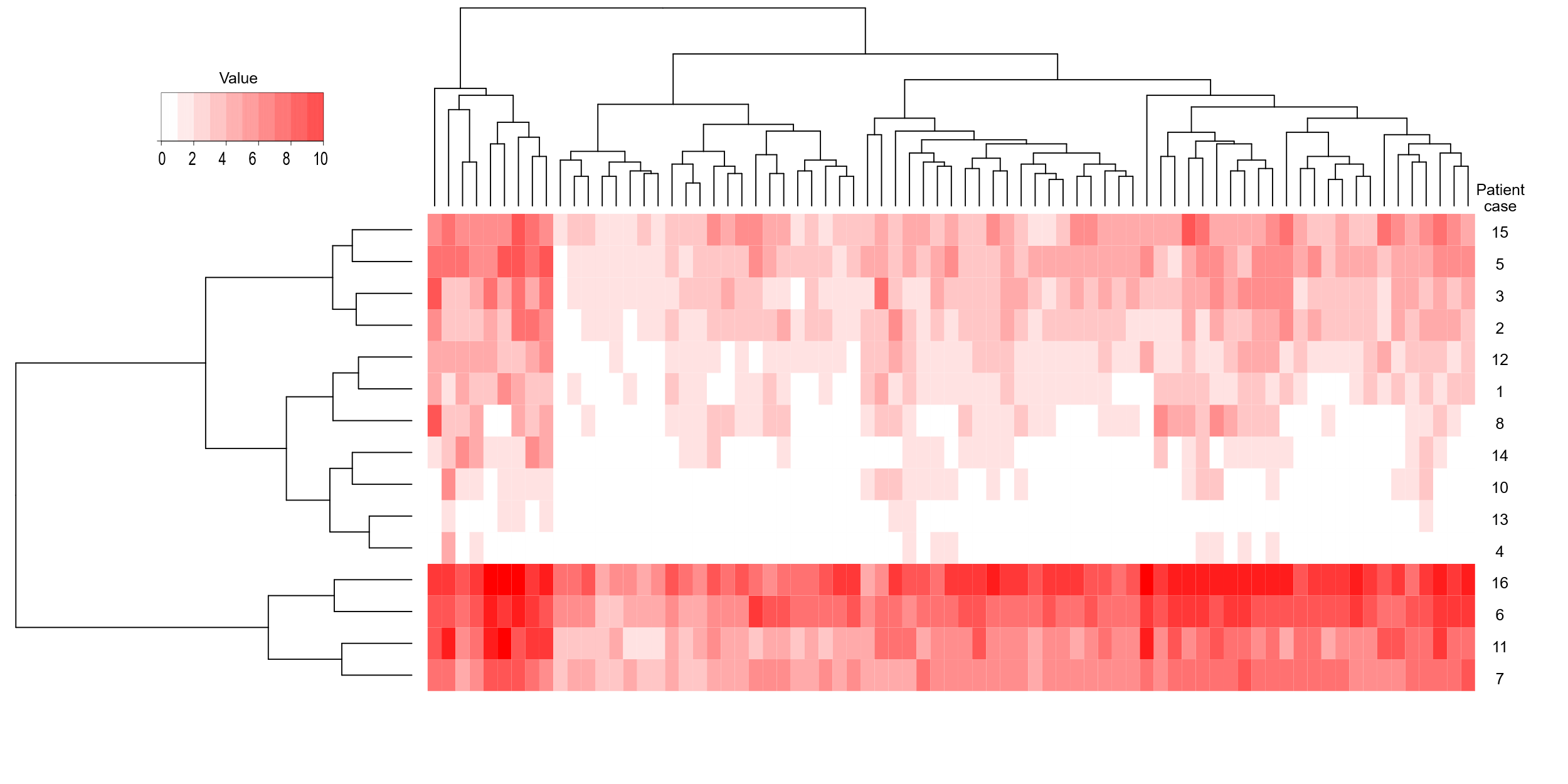


Supplementary Figure S2. A heatmap displaying all Physician Global Assessment (PhGA) values on a visual analogue scale (VAS) from 0 to 10 in whole numbers, with 0 being no disease activity and 10 being the most severe disease activity for all imaginary patient cases as assessed by all physicians.

This visualisation highlights the cohorts of patients receiving higher PhGA values and identifies physicians who consistently assign either higher or lower values. The x-axis represents all physicians, while the y-axis represents all patient cases. The colour gradient in the upper corner of the map indicates the VAS values assigned by the physicians.

| **Demographics n(%)** | **All (n=75)** | **Group A (n=8)** | **Group B (n=7)** | **Group C (n=60)** | **p-value** |
| --- | --- | --- | --- | --- | --- |
| Female | 58(77.3) | 6(75.0) | 6(85.7) | 46(76.7) | 1 |
| Not a rheumatologist/specialised in rheumatology | 11(14.7 | 2(25.0) | 1(14.3) | 8(13.3) | 0.6 |
| Physician specialising in rheumatology | 13(17.3) | 1(12.5) | 2(28.6) | 10(16.7) | 0.732 |
| Less than 5 years of experience as rheumatologist | 13(17.3) | 2(25.0) | 2(28.6) | 9(15.0) | 0.39 |
| 5-10 years of experience as rheumatologist | 9(12.0) | 1(12.5) | 1(14.3) | 7(11.7) | 1 |
| Over 10 years of experience as rheumatologist | 29(38.7) | 2 (25.0) | 1(14.3) | 26(43.3) | 0.258 |

Supplementary Table S2. Demographics of the physician subgroups. The physicians were divided into three groups according to the PhGA values they assigned to the sample patients. Physicians in Group A gave exceptionally high PhGA values to at least two patients with a non-inflammatory explanation for their symptoms. Group B included physicians who never assigned a PhGA value higher than 5. Group C included the remaining physicians who were thus considered to recognise the rheumatic condition from a non-rheumatic one and to use the entire VAS when assessing the PhGA. Fisher’s exact test was used to compare the proportions between the three groups, and no statistically significant difference were observed in the demographic factors.
